# Sport practice and hemorrhoidal disease: results from a self-assessment questionnaire among athletes

**DOI:** 10.1007/s00384-024-04797-3

**Published:** 2025-01-08

**Authors:** Lucia Romano, Antonio Giuliani, Federico Paniccia, Francesco Masedu, Leonardo Tersigni, Martina Padula, Renato Pietroletti, Marco Clementi, Fabio Vistoli

**Affiliations:** Department of Biotechnological and Applied Clinical Sciences, University of L’Aquila, L’Aquila, Italy

**Keywords:** Hemorrhoidal disease, Pelvic floor, Physical activity, Risk factor

## Abstract

**Background:**

Hemorrhoidal disease (HD) is a common proctologic disease. Dietary and lifestyle play a role in the genesis of the disease or in its progression to more severe forms, although the exact mechanism is still not fully understood. We performed a pilot observational cross-sectional analytical association study to evaluate the possible association between sport activities and HD.

**Methods:**

We included subjects aged 18 years old or more, competitive and non-competitive, practicing at least one sport activity, at least twice a week. Data were collected using an online questionnaire, developed on the Microsoft Teams communication platform.

**Results:**

Out of the 312 study participants, 34% reported HD. Among subjects who practiced cycling or horseback riding, 57% reported suffering from HD; among those practicing bodybuilding, 48% complained of HD. In the multivariate logistic regression analysis, age and bodybuilding practice showed a statistically significant association with HD.

**Conclusions:**

Some sport activities could play a role in the onset or worsening of HD. Our results showed a positive association between cycling, horseback riding, bodybuilding, and HD occurrence. Given the numerous health benefits of physical activity, patients should be provided with correct information regarding the practice of sports in relation to their pathology.

**Supplementary Information:**

The online version contains supplementary material available at 10.1007/s00384-024-04797-3.

## Background

Hemorrhoidal disease (HD) is one of the most common medical and surgical conditions, and it is the main reason for a visit to a coloproctologist [1, 2]. HD affects 39 to 52% of adults and represents an important clinical and social problem. In the United States, it is estimated that more than 50% of the general population over 50 years of age has experienced symptoms related to HD [3]. However, its real prevalence may be underestimated, due to patient’s embarrassment and fear, leading to frequent self-medication [4, 5]. HD-related symptoms are particularly stressful conditions and can significantly affect patients’ quality of life and daily activities, such as playing sports. In addition, also the psychological effects and the impact on social functioning must be taken into account [4, 6].

Multiple factors have been claimed to be causes of HD occurrence, but the exact pathophysiology is poorly understood. Many studies have shown a correlation between constipation, prolonged straining, obesity, and external hemorrhoids [3]. Some aspects of toilet and lifestyle habits have also been investigated, such as intense or prolonged physical exercise [7]. However, generic advice to avoid or limit sports involving intense straining (bodybuilding, high-impact aerobics, etc.) or perineal trauma (biking, horse riding, etc.) is usually given to patients suffering from HD. Otherwise, aerobic and moderate activities are frequently recommended for their effectiveness in preventing other risk factors such as weight gain, sedentary behavior, venous stasis, constipation, and stress.

To our knowledge, no study to date has methodologically evaluated the association between sport and HD. Based on these premises, we performed a pilot observational cross-sectional analytical association preliminary study with the aim of evaluating the association between physical activities and hemorrhoidal disease.

## Methods

The study group consisted of 312 participants, of which 54% were males and 45% were females. The mean age was 38 years old (range 19–79); 106 participants suffered from HD, with an overall prevalence of 34%. The prevalence of HD among male and female participants was 21.9% and 12.2%, respectively. Features of the sample, stratified by absence/presence of HD, are reported in Table 1. The control group was composed of participants who did not refer to suffering HD.

**Table 1:** Characteristics of the sample stratified by absence/presence of HD

Variable	Total (*N* = 312)	Control group (*n* = 206, 66%)	HD group (*n* = 106, 34%)	*p*-value
Age, years; mean (SD)	38 (14.5)	36 (15.0)	43 (12.3)	< 0.001*
Sex, *n* (%)				0.026**
Male	168 (53.9)	100 (48.5)	68 (64.2)	
Female	142 (45.5)	104 (50.5)	38 (35.8)	
Other	2 (0.6)	2 (1.0)	0 (0.0)	
Sport age, *n* (%)				0.115**
< 11 years	183 (58.7)	126 (61.2)	57 (53.8)	
Among 12 and 18 years	67 (21.5)	46 (22.3)	21 (19.8)	
> 18 years	62 (19.9)	34 (16.5)	28 (26.4)	
Competitive activity				0.274**
Yes	35 (11.2)	26 (12.6)	9 (8.5)	
No	277 (88.8)	180 (87.4)	97 (91.5)	
Frequency, *n* (%)				0.596**
Once or twice a week	176 (56.4)	113 (54.9)	63 (59.4)	
3 or 4 times a week	106 (34.0)	74 (35.9)	32 (30.2)	
> 4 times a week	30 (9.6)	19 (9.2)	11 (10.4)	

*Independent samples *T*-test

**χ^2^ test

A 22-item Italian language questionnaire was designed and developed by the authors using the Microsoft Teams communication platform. It was addressed to athletes, regardless of the status of HD. The estimated mean time needed to complete the survey was 2 min. The first eight questions were aimed at investigating the subject’s anthropometric characteristics, his or her habits about sport activities, and the type of sport played. Seventeen types of sports were included in the questionnaire, identified on the basis of their prevalence in the population. The answer to question number 9 (“Have you ever suffered from hemorrhoidal disease?”) defined whether the questionnaire ended or continued, with additional questions about hemorrhoidal disease. These last questions belonged to a previously validated questionnaire (SHS_HD_) [8]. All questions were set as mandatory fields with real-time validation and automated skip logic to prevent missing data and avoid illogical or incompatible responses. No randomization of items was used. Quantitative data were automatically collected by the software and exported to a tabulated format.

The online survey was made available in May 2023, and the link was sent to the scientific society of interest to coloproctologists (Italian Society of Colorectal Surgery) and disseminated to their members. We included subjects aged 18 years old or more, currently practicing at least one sport activity, at least twice a week, or subjects who have practiced in the past at least one sport activity and have stopped it for no more than 3 years before filling off the questionnaire. We excluded patients with a history of proctological surgery or collagen diseases. The sports included in the study were grouped as shown in Table 2, based on the technical similarities among the activities.

**Table 2:** Included sport grouping

Sport	Code
Bodybuilding	1
Cycling/riding	2
Climbing/dance/athletics	3
Running	4
Padel/tennis/volleyball/basketball/rugby/soccer	5
Trekking/alpine skiing/Nordic skiing	6
Multiple sports	7
Other	0

Participation was entirely voluntary with no compensation offered. Informed consent was obtained from all those agreeing to complete the survey. Proprietary survey software and local servers were used to ensure data protection. The fully deidentified dataset was kept on password-protected computers. This study obtained approval from the local ethics committee (Prot. n. 678, 04.01.2023).

### Statistical analysis

Assuming a 5% alpha level, 80% power, and a large Cohen effect size equal to 0.8, we estimate, given balanced groups, a sample size of about 50 subjects per group.

Sample frequency distributions for the involved variables were calculated along with descriptive sample statistics of centrality and dispersion, median and interquartile range, or, where necessary, mean and variance. The statistical association between the occurrence of hemorrhoidal disease and sport activities was preliminarily evaluated by univariate analysis. A multivariate logit score assessed the predictive weight of sport activity on the HD occurrence, coding each sport type with an indicator variable, and testing its fitting using the likelihood ratio statistics. Statistical analysis was carried out using the statistical software R (R version 4.2.0. Copyright (C) 2022 The R Foundation for Statistical Computing).

## Results

In the present pilot study, a total of 312 participants were involved with a response rate of 100%. Statistically significant differences between groups (control group and HD group) emerged in the univariate analysis regarding age, sex, and sport category (Tables 1 and 3), while no significant difference was reported regarding the age of starting of sport activity (“sport age”), agonism, and frequency of training. In detail, among subjects who practiced cycling or horseback riding, 57% reported suffering from HD; among those who practiced bodybuilding, 48% reported HD (Table 3), with a statistical significance that was confirmed by data analysis in males, while it was lost within the female sex (Table 4).

**Table 3:** Prevalence of HD by sport category

HD	Sport category (%)								*p-*value
	0	1	2	3	4	5	6	7	
No	71.3	52.2	42.9	66.7	77.3	55.9	85.7	64.9	0.039*
Yes	28.7	47.8	57.4	33.3	22.7	44.1	14.3	35.1	

* χ2 test

**Table 4:** Prevalence of HD by sport category, stratified by sex

HD	0	1	2	3	4	5	6	7	*p-*value
Sport category (%), males									
No	64.3	28.6	44.4	50.0	87.5	51.8	100.0	60.7	0.006*
Yes	35.7	71.43	55.56	50.00	12.50	48.15	0.00	39.29	
Sport category (%), females									
No	73.4	88.9	33.3	80.0	71.4	71.4	62.5	77.8	0.734*
Yes	26.9	11.11	66.67	20.00	28.57	28.57	37.50	22.22	

*χ^2^ test

From the multivariable logistic regression analysis (Table 5), age (OR = 1.03, 95% CI 1.01–1.05) and bodybuilding practice (OR = 2.88, 95% CI 1.05–7.90) had a statistically significant association with the occurrence of HD. In contrast, the category including trekking, alpine skiing, and Nordic skiing seemed to reduce the risk of disease onset (OR = 0.24, 95% CI 0.06–0.95).

**Table 5:** Multivariable logistic regression analysis

Variable	Estimate	OR	95% CI	*p*-value
Intercept	− 2.17	0.11	0.34–0.38	< 0.001
Age	0.03	**1.03**	1.01–1.05	**0.001**
Sex	− 0.33	0.70	0.39–1.25	0.230
Sport category 1 2 3 4 5 6 7	1.05 0.89 0.83 − 0.36 0.70 − 1.40 0.14	**2.88** 2.39 1.88 0.69 2.01 **0.24** 1.15	1.05–7.90 0.82–7.02 0.40–8.85 0.22–2.17 0.80–5.04 0.06–0.95 0.55–2.38	**0.039** 0.111 0.423 0.530 0.137 **0.042** 0.713
Training frequency	0.01	1.01	0.69–1.97	0.950
Competitive activity	− 0.69	0.52	0.20–1.24	0.152
Starting age	0.14	1.13	0.90–2.17	0.469

Among people who reported having symptoms related to hemorrhoidal disease, 19% felt that these symptoms moderately or completely affected their physical activity, and 24% of them reported that they had to reduce the intensity or frequency of their workouts. Finally, 52% of them have never undergone a specialist examination for this problem.

## Discussion

In our study, we investigated the role of some sport activities in promoting or worsening HD. The anal cushions of patients affected by HD are characterized by abnormal venous dilatation and degenerative process in the collagen fibers and fibroelastic tissues [9]; several possible risk factors have been identified (genetic predisposition, sedentary life, tight-laced clothes, climate, poor-fiber diet…) [3, 7, 9–11]. Instructions to patients published in *JAMA* [12] declare that “anything that puts pressure on the veins in the lower body can lead to hemorrhoids, including straining during a bowel movement, sitting on the toilet for long periods, constipation or diarrhea, being overweight, pregnancy, and age.” In this view, the main responsible factor seems to be the increased intra-abdominal pressure, which causes obstruction of venous outflow, resulting in engorgement of the hemorrhoidal plexus [3]. Moreover, increased intra-abdominal pressure could also weaken pelvic floor structures.

In our study, we reported a 34% prevalence of HD among competitive and non-competitive athletes. In the general population, the estimated prevalence rate of HD seems to be 4.4% [13–15], although it could be underestimated because patients have a tendency to use self-medication rather than seek medical attention. In the UK, hemorrhoids were reported to affect 13–36% of the general population [3, 9]. In another study [16], the prevalence rate was found to be 13.1%; these results are consistent with studies conducted in Israel [17] and Korea [18] with 16% and 14.4% prevalence, respectively. Other studies from Australia and Egypt reported the prevalence to be 38.9% and 18%, respectively [15, 19]. In this view, according to our results, it seems that athletes are more likely to have HD as compared to the general population. In particular, bodybuilding practice had a statistically significant association with the occurrence of HD. As previously stated, this could be linked to increased intra-abdominal pressure, causing both a distal displacement of anal cushions and obstruction of venous outflow. This may result in engorgement of hemorrhoidal cushions, and degeneration of elastic and connective fibers [20] of the pelvic floor. In fact, although athletes may have a strong pelvic floor, the pelvic muscles may still be too weak or not coordinated in counteracting the intraabdominal pressure during strenuous activities [20].

Sport activities may increase abdominal pressure by two exercise modalities: strenuous strength training, such as weightlifting, and high-impact activities, such as jumping and running. The former is characterized by short-duration bursts of impact with high increases in abdominal pressure [21]; the latter is associated with a high number of impacts from ground reaction forces. Hay [22] estimated that during running vertical ground reaction, forces would be 3–4 times the body weight, and during jumping, they would be 5–12 times.

Previous authors underlined the role of sport activities on some diseases of the pelvic floor, in particular on urinary incontinence. In fact, its prevalence resulted in being more common among exercising women who performed higher impact activities [20, 23–26]. Otherwise, moderate physical activity, such as walking, may decrease the risk of urinary incontinence. Our results also show that trekking, alpine skiing, and Nordic skiing seemed to reduce the risk of disease onset.

As far as anal incontinence is concerned, high-level sports appear to be a significant independent risk factor according to Vitton et al. [27], while other studies are inconsistent in their conclusions on this topic [28–30].

We found that, in people who reported symptoms of HD, 19% felt that this moderately or completely affected physical activity and 24% of them complained that they had to reduce the intensity or frequency of their workouts.

Physical activity has been shown to have many benefits, including improved mental health, and well-being [31]. The need to stop or reduce it, therefore, could impact quality of life. This is the main reason why it is relevant to establish accurate and scientifically based guidelines on sport activities and HD.

## Conclusion

Some activities could play a role in the onset or worsening of hemorrhoidal disease. Our results showed a positive association between cycling, horseback riding, bodybuilding, and HD occurrence. In contrast, the sport category including trekking, alpine skiing, and Nordic skiing seemed to reduce the risk of disease onset. Given the numerous health benefits of physical activity, no one should be recommended to stop exercising. However, patients should be provided with correct information and advice regarding the practice of sports in relation to their pathology.

## Supplementary Information

Below is the link to the electronic supplementary material.

Supplementary file1 (PDF 326 KB)

Supplementary file2 (PDF 390 KB)

## Data Availability

No datasets were generated or analysed during the current study.
