# Supplementary material for: Sport practice and hemorrhoidal disease: results from a self-assessment questionnaire among athletes
Source: Int J Colorectal Dis. 2025 Jan 8;40(1):8. doi: 10.1007/s00384-024-04797-3 (PMC11706884; doi:10.1007/s00384-024-04797-3)
Supplement: Supplementary file 1 — Supplementary file1 (PDF 326 KB) [file 384_2024_4797_MOESM1_ESM.pdf]

SPORTS AND HEMORRHOIDAL DISEASE
University of L'Aquila
Department of Biotechnological and Applied Clinical Sciences

* answer required

1. Information and consent to participation and data processing *.
The time required to complete the questionnaire is about 1 minute.
Participation in the questionnaire is voluntary; you may stop the questionnaire at any question, without submitting a response.
The collected data will be used in an anonymous and aggregated form exclusively for the purpose of publication of scientific articles for national and international journals.
By completing the questionnaire and submitting your responses you consent to the collection, analysis and potential publication of your data in an anonymous and aggregated form.
You understand that no personal information will be collected that could lead to your identification.
The study has been approved by the University Ethics Committee (Prot. No. 678, 04/0J/2023)
For more information: antonio.giuliani@univaq.it

- I agree
- I do not agree

1. you declare that you are of legal age *
   - yes
   - no

2. specify your age *

The value must be a number

3. sex
   - M
   - F
   - other

4. Do you suffer from any medical condition? (If yes, please indicate it in the text bar below)

5. Sports played *

- o running
- o cycling
- o body building
- o soccer
- o contact sports-martial arts
- o trekking
- o climbing
- o basket
- o volleyball
- o pentathlon/triathlon
- o athletics
- o alpin ski/nordic ski
- o padel
- o tennis
- o riding
- o rugby
- o dance
- o other

6. How often do you train? *

- o 1-2 times a week
- o 3-4 times a week
- o more than 4 times a week

7. Are you a competitive sportsman? *
(Registered with relevant sports federation)

- o yes
- o no

8. At what age did you start playing sports? *

- o childhood (before age 11)
- o adolescence (12-18 years)
- o adult age (from 18 years)

9. Have you ever suffered from hemorrhoidal disease? *

- o yes
- o no

10. Is your quality of life affected by hemorrhoids? *

not at all slightly moderately completely

11. When did you start suffering from symptoms related to hemorrhoids? *

- o before I started playing sports
- o after starting to play sports

12. At what age did you start having these symptoms? *

- before the age of 20
- between the ages of 20 and 40
- between the ages of 40 and 60
- after the age of 60

13. How often do you have pain or discomfort? *

- never
- at least once a year
- at least once a month
- at every defecation

14. How often do you have bleeding? *

- never
- at least once a year
- at least once a month
- at every defecation

15. How often do you feel hemorrhoid leakage? *

- never
- at least once a year
- at least once a month
- at every defecation

16. Have you ever had to manually reposition hemorrhoids inside? *

- never
- at least once a year
- at least once a month
- at every defecation

17. If you have symptoms, when do they occur? *

- at rest
- after physical activity
- both

18. How much do hemorrhoids affect your sports performance? *

not at all   slightly   moderately   completely

19. Have you ever had to stop exercising because of symptoms related to hemorrhoids? *

- no
- I have reduced the intensity
- I have reduced the frequency of training
- I changed sport
- I stopped playing sport

20. If you have changed sports, indicate which sport and give reasons for your choice

21. Have you ever had a specialist (proctological) examination? *

- yes
- no

22. Have you ever had therapy? *

- no
- yes, medical
- yes, surgical
